# Improve the Performance of Mechanoelectrical Transduction of Ionic Polymer-Metal Composites Based on Ordered Nafion Nanofibres by Electrospinning

**DOI:** 10.3390/polym10070803

**Published:** 2018-07-21

**Authors:** Yang Zhao, Jiazheng Sheng, Di Xu, Minzhong Gao, Qinglong Meng, Dezhi Wu, Lingyun Wang, Wenlong Lv, Qinnan Chen, Jingjing Xiao, Daoheng Sun

**Affiliations:** 1 Department of Mechanical and Electrical Engineering, Xiamen University, Xiamen 361102, China; zhaoy@xmu.edu.cn (Y.Z.); shengjiazheng@stu.xmu.edu.cn (J.S.); axudi@stu.xmu.edu.cn (D.X.); mengqinglong@stu.xmu.edu.cn (Q.M.); wdz@xmu.edu.cn (D.W.); wangly@xmu.edu.cn (L.W.); chenqinnan@xmu.edu.cn (Q.C.); sundh@xmu.edu.cn (D.S.); 2 School of Aerospace Engineering, Beijing Institute of Technology, Beijing 100081, China; j.gmz@163.com; 3 Pen-Tung Sah Institute of Micro-Nano Science and Technology, Xiamen University, Xiamen 361005, China; lwl1980@xmu.edu.cn

**Keywords:** Nafion, ionic polymer–metal composite, mechanoelectrical transduction, electrospinning, nanofibre

## Abstract

An ionic polymer–metal composite (IPMC) is a kind of soft material. The applications of IPMC in actuators, environmental sensing, and energy harvesting are currently increasing rapidly. In this study, an ordered Nafion nanofibre mat prepared by electrospinning was used to investigate the characteristics of the mechanoelectrical transduction of IPMC. The morphologies of the Nafion nanofibre mat were characterized. The proton conductivity, ion exchange capacities, and water uptake potential of the Nafion nanofibre mat were compared to traditional IPMC, respectively. A novel mechanism of Nafion nanofibre IPMC was designed and the open circuit voltage and short circuit current were measured. The maximum voltage value reached 100 mv. The output power was 3.63 nw and the power density was up to 42.4 μW/Kg under the load resistance. The Nafion nanofibre mat demonstrates excellent mechanoelectrcical transduction behavior compared to traditional IPMC and could be used for the development of self-powered devices in the future.

## 1. Introduction

**Figure 1:**
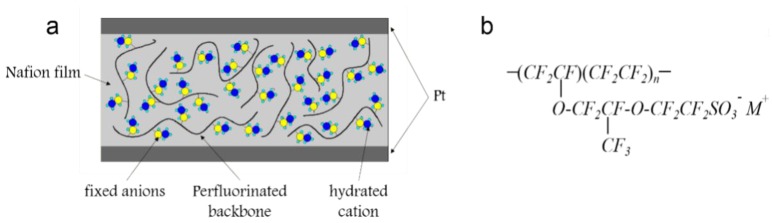
Schematic diagram of IPMC (**a**) “Sandwich” Structure (**b**) Molecular Formula of Nafion.

Ionic polymer–metal composites (IPMCs) are a new kind of smart material. They consist of one proton exchange mat layer and two metal electrodes on both sides. The structure of IPMC is similar to a “sandwich”, as shown in Figure 1a. When a DC voltage difference is applied on both ends of the electrodes, IPMC generates mechanical bending in the direction of the anode [1]. On the contrary, if a mechanical deformation occurs in IPMC, a voltage is generated on both sides of the electrodes. Therefore, IPMC is a promising class of soft electroactive polymers that can be utilized as actuators [2,3], sensors [4,5], or energy harvesters [6,7] due to its mechanoelectrical transduction. Although IPMCs used as energy harvesters allow for energy conversion in a broad frequency range, especially in a low frequency, the electrical output is small [8,9,10,11]. It greatly limits the application of IPMC energy harvesting in practical use.

Recently, most work on ionic polymers has tried to model the behavior, improve the performance, and apply them in different fields. Wang et al. proposed a double-chamber valve-less pump actuated by a cantilever ionic polymer-metal composite, which validates the suitability for use in drug delivery applications [12]. Horiuchi et al. demonstrated a new voltage-controlled accommodating IOL consisting of an IPMC actuator to change the lens’ focus applied in the eye [13]. Hong et al. investigated electrochemical and morphological characteristics of IPMCs by varying the morphology of their metal composite component [14]. Dominik et al. investigated the sensing capabilities of IPMC as a displacement sensor [15]. Two mechanoelectrical transduction principles have been reported: ion transport in the constitutive mat [16,17,18,19] and an electrostatic effect [16,20] resulting from cation and anion location forming a cluster. The researchers generally agree with the theory of the “ion cluster network model” in Nafion [21,22]. Ma et al. adjusted the nano-scale structure of the self-assembled proton conductive channels by enlarging the nano-scale pore size within the macromolecules. The hybrid hyperbranched polyamide proton exchange mat (PEM) exhibits promising application potential [23]. Xia et al. performed nanoindentation tests to study the influence of the temperature condition on the mechanical properties of the Proton Exchange Mat. The results indicate that both hardness and elastic modulus show non-monotonic transition with the increase of temperature [24]. Tiwari et al. presented a physics-based mechanoelectric model that takes into account material properties, electrostatic phenomenon, and ion transport in the IPMC to study the Mechanoelectric transduction in an ionic polymer-metal composite [25]. Tiwari et al. studied different parameters in the mechanical domain and the electrical domain to investigate the applicability of IPMC as an energy harvester in lower frequency regions [26]. Morohoshi et al. established methods to evaluate key properties such as water diffusion, gas permeability, and mechanical strength based on coarse-graining models, and an optimal design of the polymer structure could be established by the method [27]. Bing Guo et al. synthesised a hydrophilic polymer shell on a silica microsphere template and the subsequent removal of the template by etching to obtain the tiny hydrophilic hollow chamber, because it is critical to retaining moisture and facilitating proton transfer in the composite mats [28].

The electrical output performance of IPMC is determined by characteristics of mechanoelectrical transduction of Nafion. Figure 1b shows the molecular formula of Nafion. Due to the hydrophobic nature of the perfluorinated backbone and the hydrophilicity of the side chain containing sulfonic acid groups in the Nafion mat, the Nafion mat exists as microphase separation, forming a hydrophilic ion channel. The sulfonic group is immobilized on the side chain, and only hydrated cations can move freely. When mechanical deformation of IPMC occurs, the volume of the compression side is reduced and that of the stretch side is increased. This leads to the movement of the hydrated cations in IPMC. Owing to changes in the local concentration in the Nafion mat, an electrical potential difference forms on both sides of the IPMC. However, the ion channel is random, irregular, and without a specific orientation in the Nafion mat. When mechanical deformation of IPMC occurs, the transfer of hydrated cations requires a long transmission path and overcomes a large resistance from one side to the other. Up to now, fewer researchers have concentrated on the structure to improve the electrical output by studying ion transfer in the Nafion mat. Muchao Qu et al. studied the electrical conductivity of melt spun composites consisting of PMMA containing both aligned carbon fibers and carbon black, which showed a decreased tensile strength and an increased E-modulus with the addition of fillers [29]. Qiang Chen et al. modified bulk metallic surfaces by the electrophoretic deposition of short phosphate glass fibers to improve bone-to-implant bonding [30]. Yahong Li et al. presented a combined electrospinning, ultrasonication adsorbing, and bobbin winder technique by electrospun thermoplastic polyurethane fiber yarns successively decorated with multi-walled carbon nanotubes and single-walled carbon nanotubes to obtain highly conductive and stretchable yarns [31]. This work aims to discuss the oriented transfer of ions by using an ordered Nafion nanofibre mat which has not been considered previously.

In this paper, an ordered Nafion nanofibre mat is proposed to improve the mechanoelectrical transduction by electrospinning. The ordered Nafion nanofibre was collected by a rotated roller. The mechanism of IPMC based on the Nafion nanofibre mat was fabricated to test the characteristics of mechanoelectrical transduction. The surface morphologies of the Nafion nanofibre were characterized. The water uptake potential, ion exchange capacity, and proton conductivity were compared with traditional IPMC. The open circuit voltage and short circuit current of IPMC based on the Nafion nanofibre mat were measured under different thicknesses and different loads. The electrical output of IPMC based on the Nafion nanofibre mat under different load resistances was also tested.

## 2. Experimental

### 2.1. Materials

DuPont^TM^ Nafion PFSA polymer dispersions were made from chemically stabilized perfluorosulfonic acid/PTFE copolymer in the acid (H^+^) form that were used to electrospin the ordered nanofibre mat. The Nafion dispersion of DE2020 (the polymer content is 20–22%) was supplied by DuPont Co., (Wilmington, DE, USA), the Poly ethylene oxide (PEO: Molecular weights of 5,000,000) was purchased from Changchun Huayu Fine Chemical Co. Ltd., (Changchun, China), and absolute ethyl alcohol was obtained from Shantou Dahao Fine Chemical Co. Ltd., (Shantou, China). The PET film with a thickness of 0.1 mm was purchased from Suzhou Aiqiu Electronics Co., Ltd., (Suzhou, China). The silicone elastomer (PDMS) was obtained from Dow Corning Co. Ltd., (Midland, MI, USA). The PDMS was added with a curing agent (ratio 10:1) and was fully mixed with manual stirring for 3 min, and then the mixture was vacuumed for 10 min to remove the bubbles. It was prepared for future use.

### 2.2. Fabrication of Ordered Nafion Nanofibre Mat

The mat of the ordered Nafion nanofibre was fabricated by the electrospinning method. Because the Nafion dispersion could not be electrospun, the PEO was added to Nafion dispersion to improve the electrospinning ability. The weight of nafion was 2 g. The weight ratio of nafion dispersion and PEO was 99:1. The proportion of solvent alcohol and deionized water was 1:1. The mixed solution was stirred by a magnetic stirrer for 4 h. Since PEO has no contribution to the proton conductivity of nanofibres, it was added to Nafion solution only to improve the solution viscosity and molecular entanglement force. At the same time, PEO was a useless impurity for Nafion nanofibres, so high purity Nafion solution was chosen for electrospinning. However, little PEO content could result in the poor quality of nanofibres, such as bead-on-nanofibres, and even make Nafion unable to be electrospun. Finally, 1% content of PEO was selected and high quality nanofibres were obtained.

**Figure 2:**
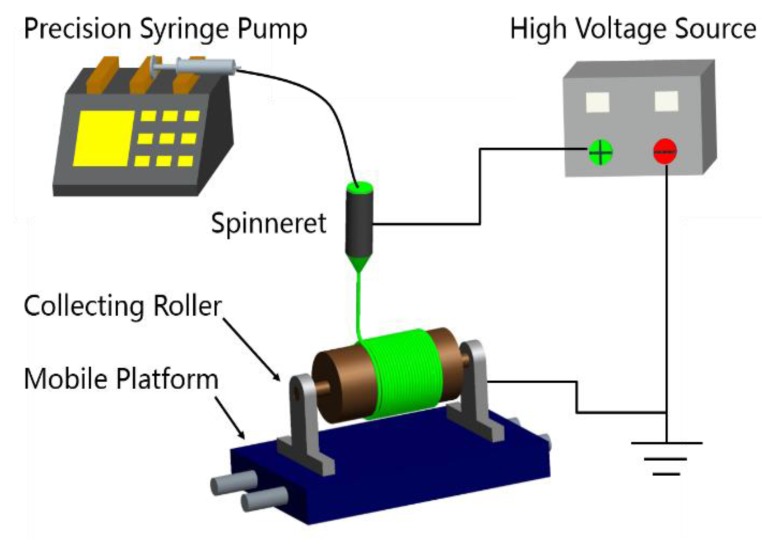
Schematic diagram of the rotating roller electrospinning.

The electrospinning apparatus was schematically shown in Figure 2. The precision syringe pump provided a steady and continuous supply of solution for the syringe. The high-voltage power supply was applied to the nozzle to supply an electric field force for electrospinning. A rotating roller was used to collect nanofibres and the roller was mounted on the mobile platform moving laterally, so that the collected nanofibre mat had a certain width and uniform thickness. The related parameters of electrospinning are summarized in Table 1.

### 2.3. Measurements and Characterizations of Nafion Nanofibre Mat

Water uptake potential: The WUP was determined by the difference between the weight of the vacuum-dried mat and the fully water-equilibrated mat. The mat was placed in deionized water for 24 h. The mat was quickly wiped with absorbent paper to remove the surface water, and the weight of the sample was recorded. Then, the sample was placed in a vacuum dried box to remove residual solvent or water and the weight was recorded for 12 h. The water uptake potential of the mat was calculated by the following equation:

(1) $$\;\mathrm{WUP}=\frac{W_{wet}-W_{dry}}{W_{dry}}\times 100\%\;$$

where $W_{wet}$ and $W_{dry}$ denote the weight of the wet and dried mats, respectively. 

Ion exchange capacity: The IEC was the capacity of ion exchange in the mat. First, the mat was dried in a vacuum dryer for 12 h and then immersed in 10 mL 0.1 mol/L NaOH solution for 12 h. Then the mat was taken out and the phenolphthalein solution was dropped in NaOH solution. Finally, 0.1 mol/L HCl solution was added to the extracted solution until it became neutral. The IEC value was calculated with the amount of the additive HCl solution. The IEC value was determined with the following equation:

(2) $$\;\mathrm{IEC}=\frac{V_{NaOH}\times N_{NaOH}-V_{HCl}\times N_{HCl}}{weight\;of\;dry\;membrane}\;$$

where *V* denotes the volume of the solution and *N* denotes normality.

Proton conductivity: Proton conductivity of the mat was measured between two copper electrodes at both ends by connecting it to an impedance analyzer. The input frequency was 100–110 MHz and the amplitude was set at 5 mV. The equation used for calculating conductivity is given below.

(3) $$\;\delta =\frac{l}{R_{b}\times A}\;$$

where δ is the proton conductivity, *l* is the length of the film between the electrodes, $R_{b}$ is the bulk resistance of the mat, and *A* is the cross sectional area of the mat.

Scanning electron microscopy: Scanning electron microscopy (SEM) images of surface morphology were taken to observe ordered Nafion nanofibre mats. SEM images of all samples were obtained by using an SU-70 thermal field emission scanning electron microscope (Hitachi Inc., Tokyo, Japan). The surfaces of the Nafion nanofibre mats and other structures were observed.

### 2.4. Mechanoelectrical Transduction Mechanism of the Nafion Nanofibre IPMC

**Figure 3:**
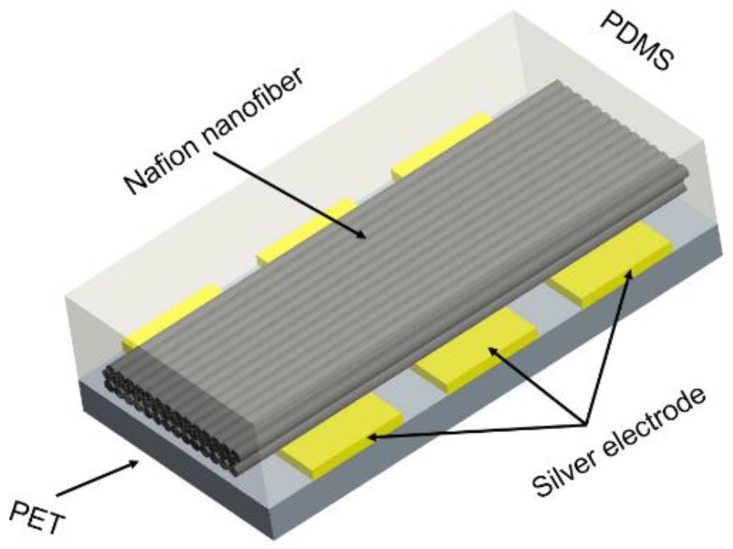
Schematic of the experimental mechanism of the ordered Nafion nanofibre mat.

Generally, ionic polymer-metal composites were fabricated by electroless plating Pt electrodes on both sides of the Nafion-117 film, just like a “sandwich” structure, but this structure was not suitable for Nafion nanofibre mats. An experimental mechanism schematic which was used to test the characteristics of mechanoelectrical transduction based on ordered Nafion nanofibre mats is shown in Figure 3. The Nafion nanofibres mat was fabricated by electrospinning. The substrate was a PET film. The electrodes were evenly coated on the substrate by silver paste. The size of the silver electrodes was 25 mm × 5 mm and the spacing between them was 2 mm. Then, it was heated at 60 °C for 30 min to solidify it. The Nafion nanofibre mat was put on the electrodes along the direction perpendicular to the electrodes. The PDMS was directly poured onto the surface of the nanofibre mat and placed for 30 min to fully fuse the nanofibers with PDMS. Then, the sample was placed in a drying oven and heated for three hours at 70 °C, which completely solidified the PDMS. Finally, the mechanoelectrical transduction mechanism was soaked in the saturated solution of LiCl for 12 h to make the nanofibres fully exchange the cations of Li^+^.

**Figure 4:**
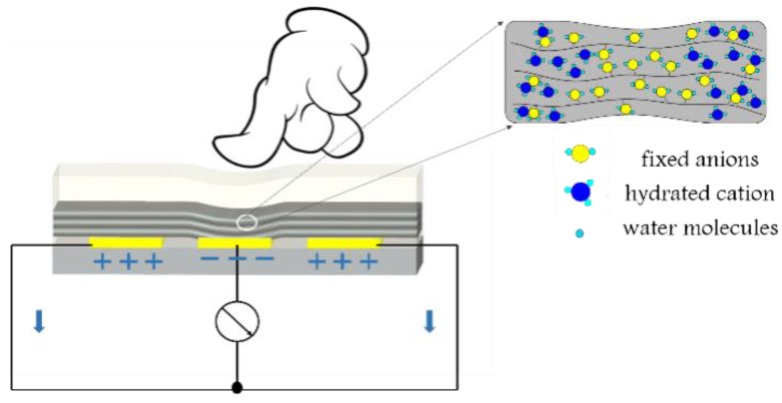
Schematic of the mechanoelectrical transduction process.

The schematics of mechanoelectrical transduction based on the ordered Nafion nanofires mat are illustrated in Figure 4. Under the state of no load, the hydration cations were evenly distributed in Nafion nanofibres due to the effect of static electricity. When applying the pressure in the middle of the Nafion nanofibre IPMC, the PDMS layer exhibited plastic deformation. Meanwhile, the deformation was transferred to the Nafion nanofibres and resulted in the move of hydration cations to both sides along the direction of the nanofibres. A voltage difference was produced between the intermediate electrode and the electrodes of both sides due to fixed anions and moving hydrated cations. When the electrodes were connected to the wire, the voltage and current signal could be measured.

## 3. Results and Discussion

### 3.1. Morphology of Ordered Nanofibre Mat

**Figure 5:**
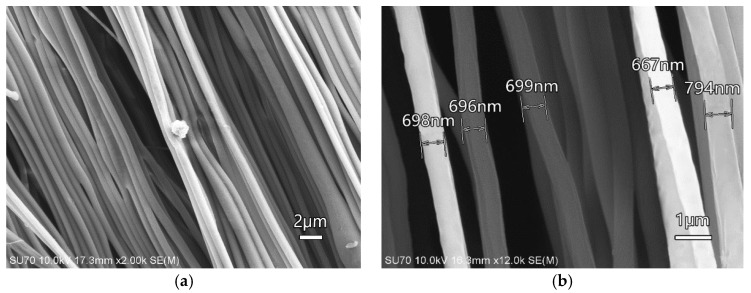
(**a**) SEM image of the ordered nanofibre mat and (**b**) the diameter of Nafion nanofibres.

Ordered Nafion Nanofibres were produced by electrospinning under high voltage. The nanofibre mat was collected by using a rotated roller. It took 36 h to obtain a nanofibre mat with the dimensions of 300 μm in thickness and 20 mm in width. The surface morphology of the nanofibre mat is shown in Figure 5a. It can be seen that the ordered Nafion nanofibres have good directionality and are arranged closely under the collection of the rollers. The diameter of Nafion nanofibres was between 600 nm and 800 nm and the nanofibres presented good homogeneity, as shown in Figure 5b. Considering the PDMS was hydrophobic, the quality of the mat in air was compared with the mat immersed in deionized water. The quality of the mat was increased after immersing it in the deionized water. It could be concluded that PDMS does not affect the water absorbability of the nanofibres.

### 3.2. WUP, IEC and Proton Conductivity

**Figure 6:**
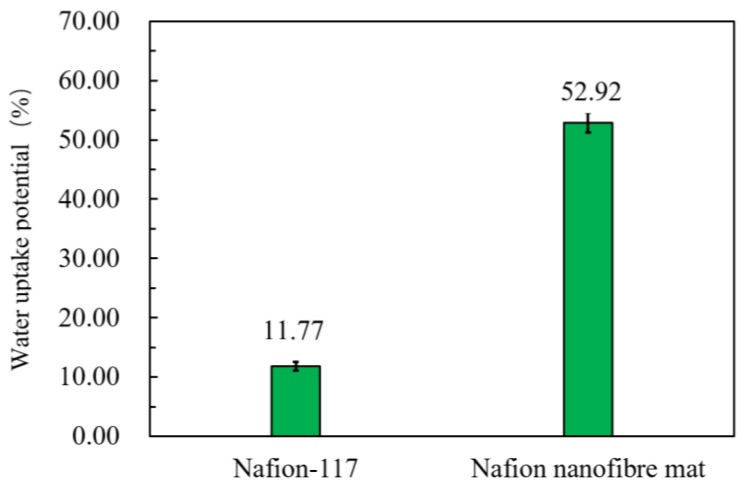
WUP of Nafion-117 and the nanofibre mat.

**Figure 7:**
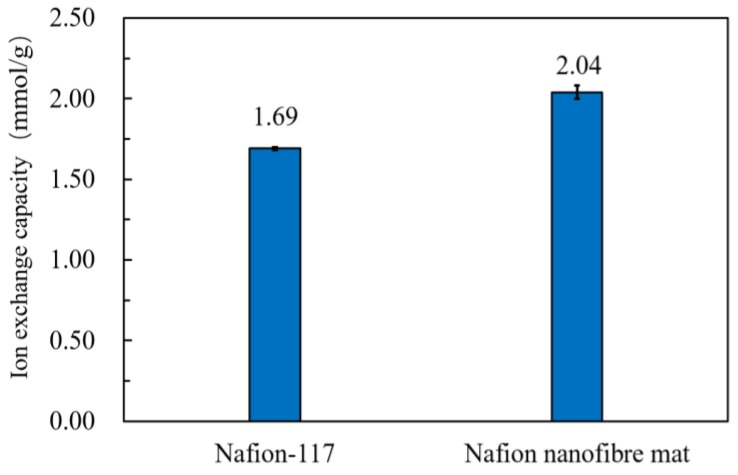
IEC of Nafion-117 and the nanofibre mat.

The properties of the sample mat are summarized in Table 2. The size of the sample was 15 mm in length and 10 mm in width. The data in Table 2 was the average value of five experiments. The WUP of the Nafion nanofibre mat was greater than the commercial film of Nafion-117. As shown in Figure 6, the WUP of the Nafion nanofibre was up to 52.92%, due to the large porosity between nanofibres that can preserve the water molecules well. In addition, the nanofibre had a large specific surface area, which ensured that the hydrophilic ion cluster made full contact with water in the nanofibre. The IEC of the Nafion nanofibre mat was higher than Nafion-117. As shown in Figure 7, the IEC of the Nafion nanofibre mat was up to 2.04, which indicates that the amount of hydrophilic ionic cluster in the Nafion nanofibre mat was more than that of Nafion-117, and the cation in the Nafion nanofibre mat was more than that of Nafion-117, meaning that it would generate more electrical output. The proton conductivity of the Nafion nanofibre mat was 0.998 S/cm, compared with 1.24 × 10^−3^ S/cm of Nafion-117. The proton conductivity of the Nafion nanofibre mat was much greater than that of Nafion-117. This indicates that the ion channel of the Nafion nanofibre mat has a certain orientation, so that the hydrated cations can transfer easily along the direction of the nanofibre. Moreover, due to the constraints of the nanofibre boundary, it was difficult for the hydrated cations to leave the ion channel in the nanofibre and most of them could only transfer along the length direction of nanofibre, so the proton conductivity was significantly improved.

### 3.3. Characteristics of Mechanoelectrical Transduction

**Figure 8:**
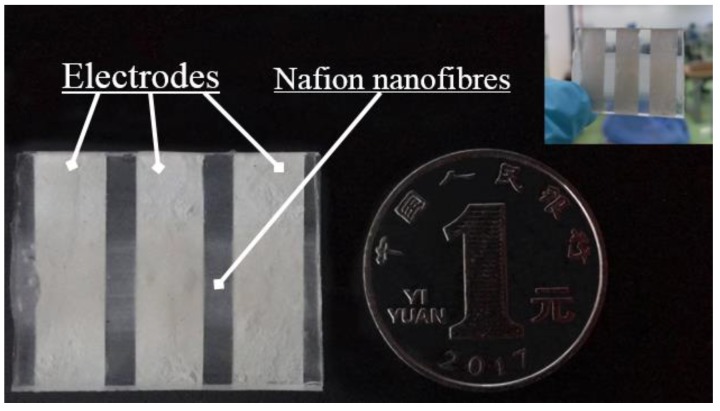
The test sample of Nafion nanofibre IPMC.

When an external force is applied on the PDMS layer of Nafion nanofibre IPMC, the Nafion nanofibre mat is compressed and deformed. This causes the cations to transfer to both sides with water molecules inside the nanofibre. A sample of Nafion nanofibre IPMC is illustrated in Figure 8. Because of the movement of hydrated cations, the concentration of the cations was nonuniform. This generates a potential difference between the center electrode and the electrodes on both sides of Nafion nanofibre IPMC. The generated potential difference prompts the movement of the external circuit electrons to form an electrical signal.

**Figure 9:**
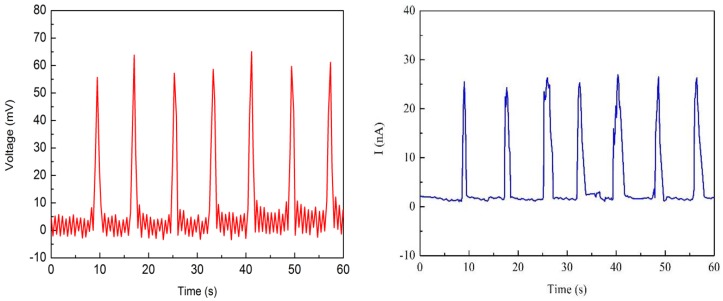
Electrical output of Nafion nanofibre IPMC (Nafion nanofibre mat 203 μm).

**Figure 10:**
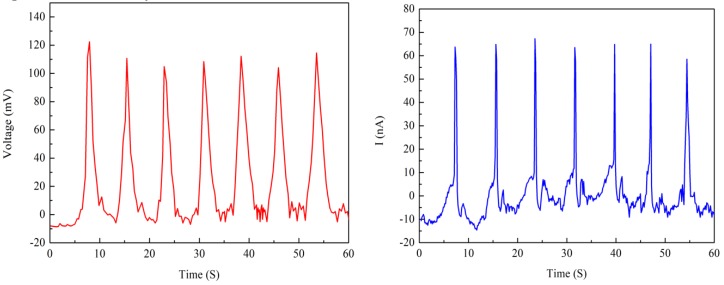
Electrical output of Nafion nanofibre IPMC (Nafion nanofibre mat 310 μm).

Two Nafion nanofibre IPMCs were fabricated to test the electrical output. The thickness of the Nafion nanofibre mat was 203 μm and 310 μm, respectively. Figure 9 and Figure 10 show the electrical output under the reciprocating load, respectively. The load was 100 N and the frequency of the load was 0.125 Hz. When measuring the output of the sample, the open circuit voltage and short circuit current could be obtained. The graphs showed that the open circuit voltage and short circuit current were in pace with the periodic changes of the load. The open circuit voltage of the 203 μm thick nanofibre mat was near 55 mv, and that which was 310 μm thick was near 100 mv. The short circuit current of the sample with the 203 μm thick nanofibre mat was near 25 nA, and that which was 310 μm thick was near 60 nA. It can be seen that the thicker the nanofibre mat is, the larger the electrical output is. As the thickness of the nanofiber mat increases, the number of cations also increases. More cations movement forms a greater potential difference between the electrodes under mechanical deformation.

Compared to traditional IPMC, the open circuit voltage of Nafion nanofibre IPMC was greatly improved. In general, the open circuit voltage of traditional IPMC is 0.1–30 mV. The voltage of Nafion nanofibre IPMC could reach 100 mV by comparison. But, compared to the short circuit current, it did not increase as expected. In traditional IPMC, the Pt electrode was fabricated by electroless plating on the surface of the Nafion-117 film. The electrode of Nafion nanofibre IPMC was fabricated by coating. Obviously, the contact resistance of electrodes of Nafion nanofibre IPMC was larger than that of traditional IPMC. Therefore, the short circuit current of the Nafion nanofibre mat did not improved remarkably.

**Figure 11:**
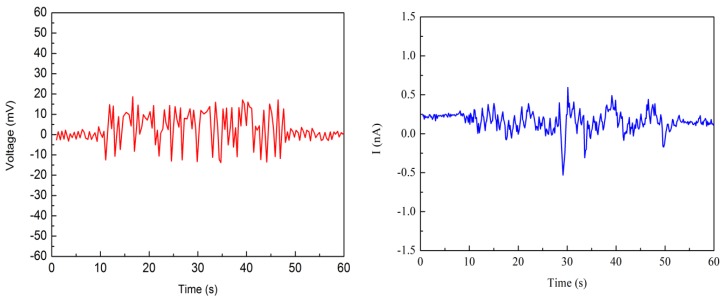
The open circuit voltage and short circuit current of Nafion nanofibre IPMC in a dry state.

The electrical output of Nafion nanofibre IPMC which was not soaked in water was also tested. The nanofibre mat was completely dry. Figure 11 shows the results of the experiment. It can be seen that there was almost no output of Nafion nanofibre IPMC in the dry state.

**Figure 12:**
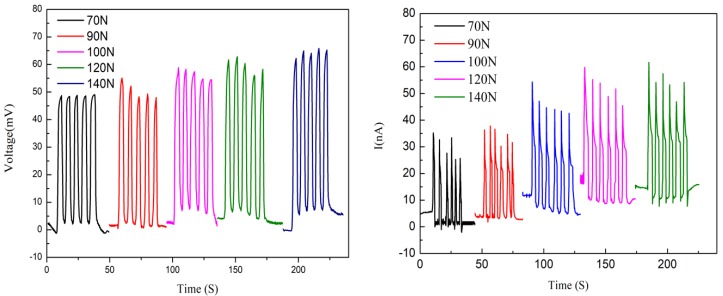
The open circuit voltage and short circuit current under different external loads.

**Figure 13:**
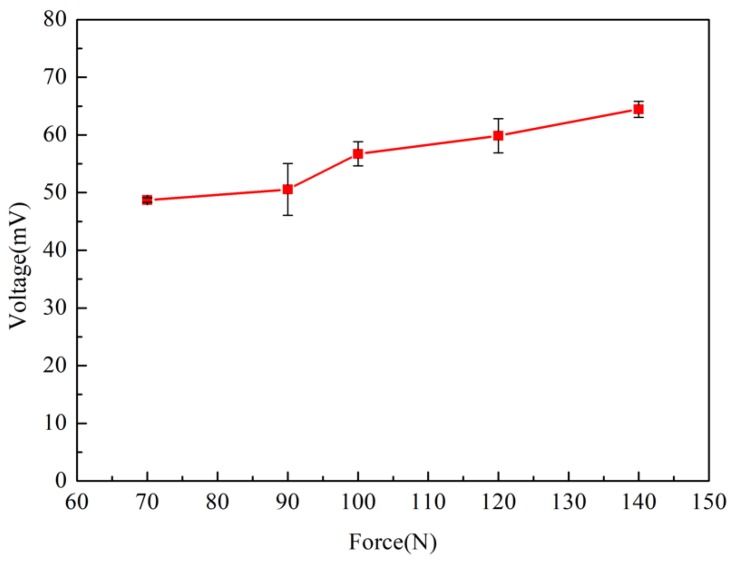
The peak output voltage under different external loads.

The electrical output under different external loads was measured. The open circuit voltage and short circuit current are shown in Figure 12, respectively. It can be seen that the open circuit voltage and short circuit current increased with the increase of the external load. But the increasing extent of open circuit voltage and short circuit current was not very high. Therefore, it can be inferred that the change of external load has little effect on the transfer of internal ions of Nafion nanofibre IPMC. Figure 13 shows the relationship between the applied force and the peak output voltage. It is a non-linear relationship.

**Figure 14:**
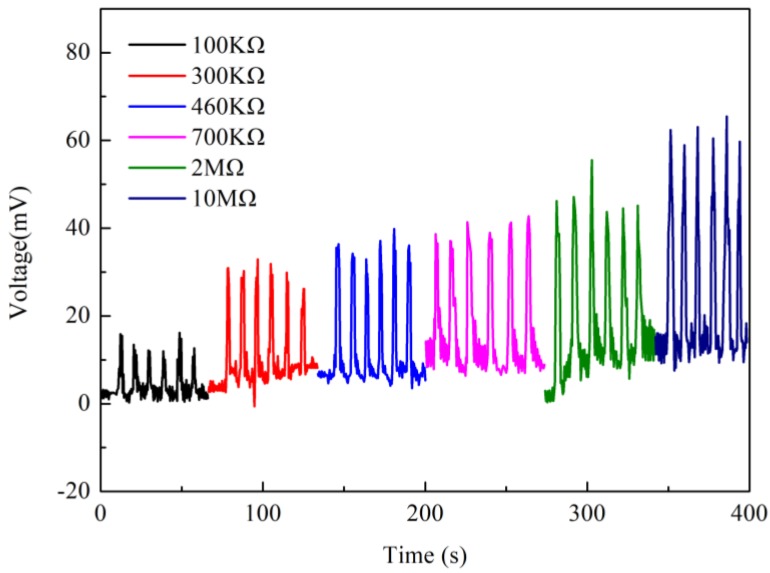
Comparison of the output voltage under different load resistances.

In order to measure the output power of Nafion nanofibre IPMC, a load resistance was put in series. Figure 14 shows the results of the output voltage under different load resistances. It can be seen that with the increase of the load resistance, the voltage also increases. The output power of Nafion nanofibre IPMC could be calculated by:

(4) $$\;P=\frac{V_{out}^{2}}{R}\;$$

where *P* is the power of Nafion nanofibre IPMC, *V_out_* is the output voltage of Nafion nanofibre IPMC, and *R* is the load resistance.

**Figure 15:**
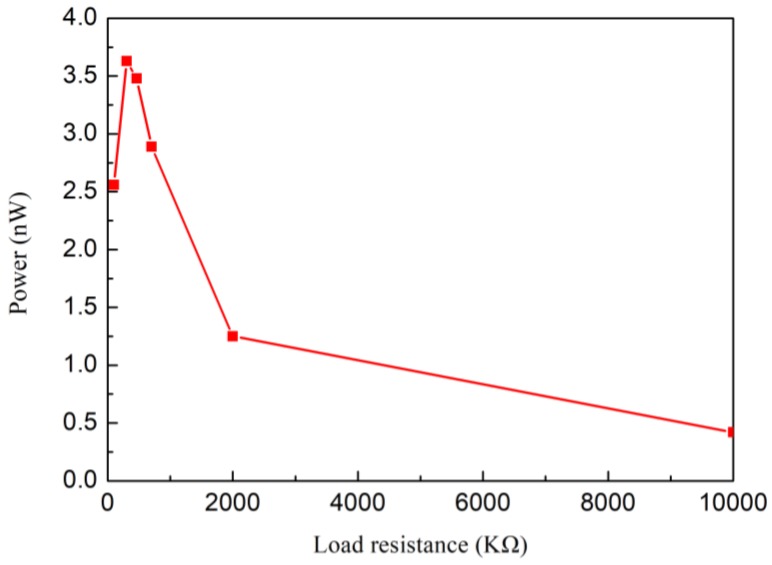
The output power under different load resistances.

Figure 15 shows the output power with different load resistances. When the external load resistance is equal to the internal resistance of Nafion nanofibre IPMC, the output power of the load resistance is the maximum. The maximum value of output power was 3.63 nW, when the resistance was 300 KΩ. It illustrates that the internal resistance of the device is near 300 KΩ. The optimal electrical output would be gained under this external load resistance. Compared to traditional IPMC, the output power of 3.63 nW was effectively enhanced. Considering the mass of the Nafion nanofibre mat (the mass of the nanofibre mat was 0.0856 g), the power density of the Nafion nanofibre IPMC would be up to 42.4 μW/Kg.

## 4. Conclusions

In this paper, a novel structure of IPMC using an ordered Nafion nanofibre mat was presented. The Nafion nanofibre IPMC was used to investigate the characteristics of mechanoelectrical transduction. The Nafion nanofibre mat was fabricated by electrospinning and collected by a rotated roller. Compared with the Nafion commercial mat, the nanofibre mat has the advantages of high water absorption and proton conductivity. Two samples of Nafion nanofibre IPMC were fabricated by using an ordered nanofibre mat with thicknesses of 203 μm and 310 μm, respectively. The electrical output of the open circuit voltage and short circuit current was compared. The maximum value of open circuit voltage was over 100 mv, which was generated by the Nafion nanofibre mat with a thickness of 310 μm. The results showed that the thicker the nanofibre mat is, the higher the open circuit voltage is. The mat in a dry state exhibited almost no electrical output. Through a series of load resistors, the output power and power density of the Nafion nanofibre IPMC were studied. Under the load resistance of 300 KΩ, the maximum of output power and the power density was 3.63 nw and 42.4 μW/Kg, respectively. The output power and power density of Nafion nanofibre IPMC was higher than traditional IPMC.

In future study, how to reduce the contact resistance of the electrodes and internal resistance to achieve greater output power will be concentrated on. Also, there is a need to study the characteristics of microcosmic movement on ion transfer inside the Nafion nanofibre IPMC.

## Figures and Tables

**Table 1:** The parameters of Nafion electrospinning.

Related Parameters	Numerical Size
Electrospinning voltage	3.0–4.0 KV
Polymer solution flow rate	160 μL/h
Inside diameter of the syringe needle	200 μm
Spinneret-to-collector distance	4 cm
Platform moving speed	60 mm/min
Roller rotation speed	200 r/min
Diameter of roller	80 mm

**Table 2:** Properties of Nafion-117 and the Nafion nanofibre mat.

	WUP (%)	IEC (mmol/g)	Proton Conductivity (S/cm)
Nafion-117	11.77	1.69	1.24 × 10^−3^
Nafion nanofibre	52.92	2.04	0.998
